# Does the availability of positron emission tomography modify diagnostic strategies for solitary pulmonary nodules? An observational study in France

**DOI:** 10.1186/1471-2407-9-139

**Published:** 2009-05-11

**Authors:** Irawati Lemonnier, Cédric Baumann, Nicolas Jay, Kazem Alzahouri, Patrick Arveux, Damien Jolly, Catherine Lejeune, Michel Velten, Fabien Vitry, Marie-Christine Woronoff-Lemsi, Francis Guillemin

**Affiliations:** 1 Equipe d'Accueil 4003, Nancy-University, Nancy, France; 2 Institut national de la santé et de la recherche médicale, Centre d'Investigation Cliniques – Epidémiologie Clinique, University Hospital, Nancy, France; 3 Faculty of Medicine, Nancy-University, Nancy, France; 4 Lorraine Laboratory of Informatics Technology Research and its Applications, Nancy, France; 5 Medical Information Department, Georges-François Leclerc Center, Dijon, France; 6 Clinical research and methodological unit, Maison Blanche Hospital, Reims, France; 7 Equipe d'Accueil 3797, Reims-University, Reims, France; 8 Institut national de la santé et de la recherché médicale, Unité 866, Faculty of Medicine, Dijon University, Dijon, France; 9 Department of Epidemiology and Public Health, Louis Pasteur University and Centre Paul Strauss, Strasbourg, France; 10 Medico-economic Evaluation Unit, Pharmacy Service, Besançon University Hospital, Besançon, France

## Abstract

**Background:**

Previous studies showed that at the individual level, positron emission tomography (PET) has some benefits for patients and physicians in terms of cancer management and staging. We aimed to describe the benefits of (PET) in the management of solitary pulmonary nodules (SPNs) in a population level, in terms of the number of diagnostic and invasive tests performed, time to diagnosis and factors determining PET utilization.

**Methods:**

In an observational study, we examined reports of computed tomography (CT) performed and mentioning "spherical lesion", "nodule" or synonymous terms. We found 11,515 reports in a before-PET period, 2002–2003, and 20,075 in an after-PET period, 2004–2005. Patients were followed through their physician, who was responsible for diagnostic management.

**Results:**

We had complete data for 112 patients (73.7%) with new cases of SPN in the before-PET period and 250 (81.4%) in the after-PET period. Patients did not differ in mean age (64.9 vs. 64.8 years). The before-PET patients underwent a mean of 4 tests as compared with 3 tests for the after-PET patients (p = 0.08). Patients in the before-PET period had to wait 41.4 days, on average, before receiving a diagnosis as compared with 24.0 days, on average, for patients in the after-PET period who did not undergo PET (p < 0.001). In the after-PET period, 11% of patients underwent PET during the diagnostic process. A spiculated nodule was more likely to determine prescription for PET (p < 0.001). Multivariate analysis revealed that patients in both periods underwent fewer tests when PET was prescribed by general practitioners (p < 0.001) and if the nodule was not spiculated (p < 0.001). The proportion of unnecessary invasive approaches prescribed (47% vs. 49%) did not differ between the groups.

**Conclusion:**

In our study, 1 year after the availability of PET, the technology was not the first choice for diagnostic management of SPN. Even though we observed a tendency for reduced number of tests and mean time to diagnosis with PET, these phenomena did not fully relate to PET availability in health communities. In addition, the availability of PET in the management of SPN diagnosis did not reduce the overall rate of unnecessary invasive approaches.

## Background

A solitary pulmonary nodule (SPN) is a single spherical lesion < 3 cm in diameter completely surrounded by lung tissue without associated atelectasis or adenopathy [1,2]. In most cases, it is detected incidentally by chest x-ray (CXR) imaging or computed tomography (CT). No data is available on its incidence in a general population, but about 1 of 500 CXR images in the United States reveals a nodule [3]. The frequency of SPN malignancy depends on the population where it is estimated, about 26% to 40% in the US [4,5] and 40% in France [6]. Therefore, SPN is often associated with lung cancer, a major public health problem, with 25,882 new cases and 27,164 deaths reported in France in 2000 [7].

The management of SPN diagnosis consists of combinations of different approaches, invasive and/or noninvasive, from the moment SPN is identified by CXR or CT until the definitive diagnosis of its nature – malignant or benign. This diagnostic approach depends on characteristics of patients (age, smoking history, antecedents of cancer) and nodules (size, location, spiculation, and calcification within the nodule). These characteristics determine the probability of malignancy [4,8]. One example of a malignant nodule is a new nodule of large size in an older patient with a heavy smoking history and CXR or CT results of a spiculated nodule pattern [9].

The newest imaging technology in nuclear medicine, positron emission tomography (PET), was introduced in oncology. It is also used in differentiating the nature of pulmonary nodules. Many studies concerning the performance of PET or PET scanning reported the sensitivity, specificity, and accuracy of this technique as 96%, 88%, and 94%, respectively, in the diagnosis of benign nodules [10-14]. Meanwhile, decision model analysis concerning the management of SPN diagnosis or studies investigating the combination of certain diagnostic tests showed that the sensitivity and specificity of the combination of examinations, with or without PET, varies depending on the type of examination performed for each patient and nodule [4,15-19]. Some techniques, such as transbronchic and transthoracic procedures, entail risk of complications (pneumothorax or haemorrhage) [18,20]. These procedures are considered unnecessary when the nodule proves later to be benign. The physician aims to prescribe the most appropriate examination to effectively diagnose the nature of the nodule, whether benign or malignant, and to avoid morbidity and/or mortality due to unnecessary invasive explorations [21]. The use of PET could reduce those risks by avoiding unnecessary explorations.

Research into the ability of PET to differentiate malignant from benign nodules have proved its good performance at the individual level compared to other imaging technologies such as CT or magnetic resonance imaging (MRI). Results of PET could change the cancer's staging or physicians' decision for further exploration in cancer management [22]. Unfortunately, few studies are available on changes in the management of SPN diagnosis that PET brings in real-life practice at a population level. Knowing whether the availability of PET contributes to altering the number of tests performed and avoiding unnecessary invasive approaches for patients with benign nodules in routine practice is important for patients and for physicians.

Therefore, we aimed to study the actual practice of managing the diagnosis of SPN before PET was introduced in France as a diagnostic test (2002–2003; before-PET) and after its introduction (2004–2005; after-PET) – a before-after study. We aimed to describe the modifications in the management of SPN diagnosis in daily practice in terms of average number of tests performed, time to diagnosis and proportion of unnecessary invasive tests performed with PET availability in the 5 northeastern regions of France. We also identified determinants of the use of PET in the after-PET period and the use of invasive approaches in both periods.

## Methods

The study was approved by the Institutional Review Board (Commission National d'Informatique et Liberté [CNIL]) in January 2002. PET was available for use in 5 regions in northeastern France (Alsace, Lorraine, Franche-Comté, Bourgogne, and Champagne-Ardenne), 18 health administrative districts covering 8,220,000 people [23], in 2003–2004. We collected data on the before-PET period between May 2002 and March 2003 and on the after-PET period between June 2004 and June 2005. The before-PET period was shorter because of the limited time before PET availability in each region.

### Study Samples

Periods of 4–8 weeks, with duration adjusted to the population size and number of CT centres in each region, were randomly sampled on the year's calendar to equally balance the number of patients included in each region. Qualified research assistants examined 31,590 CT reports, compiled by the radiologists in each centre. They examined 11,515 reports in the before-PET period and 20,075 in the after-PET period, in all (76) CT centres in the 5 regions. We identified new SPN cases from CT reports that mentioned "spherical lesion" or "nodule" or synonymous terms (including "mass" or "solitary mass", "solitary pulmonary nodule" or "SPN"), A panel of investigators verified all included cases.

We included patients who were ≥ 18 years old, had a nodule of 1 to 3 cm in diameter, were without antecedent cancer, and followed the diagnostic management in all care centres involved in the 5 regions. Patients were not included if they had multiple nodules, or who underwent the diagnostic management outside of the 5 study regions.

Studies reported that one of the major benefits of PET was a reduction in the number of patients referred for surgery. Depending on the pre-test probability of malignant disease, the use of PET decreased the performance of surgical procedures by an estimated 15% [24]. To observe a difference of at least 15% between both periods in terms of invasive approaches for patients with benign nodules and for a power of at least 80%, we doubled the number of patients included in the after-PET period. This step was necessary because the before-PET period entailed time constraints before the introduction of PET and we could include only a limited number of patients.

### Data collection

A research assistant in each region collected data on socio-demographic characteristics of patients such as age, sex, and smoking history; and nodule characteristics, such as calcification within the nodule, appearance of spiculated nodule, and mediastinal involvement or enlarged lymph nodes seen on CT. Information on examinations and treatments that were prescribed during 6-month follow-up was collected as well.

We defined the beginning of the diagnostic management as the date of the CT report identifying the nodule. We considered that the presumptive diagnosis of cancer was established when physicians who handled the management of the diagnosis decided to discontinue the diagnosis process and to begin surveillance for benign nodules or treatments for malignant nodules. We followed all patients for 6 months after the presumptive diagnosis through the hospital or the physician who was in charge of managing the diagnosis. Diagnostic management was considered specialised if performed by a radiologist, chest physician, oncologist or thoracic surgeon.

The number of tests were those prescribed by a physician for a given patient from the beginning of the diagnostic management until the presumptive diagnosis. The diagnostic management was defined as the combination of examinations in chronological order from the identification of SPN on CT up to the presumptive diagnosis of SPN. We defined invasive approaches as any combination of tests involving at least one nonimaging examination. Unnecessary invasive approaches were defined as those involving one or more invasive explorations in which the nodule was later diagnosed to be benign.

### Statistical Analysis

We used the chi-square test to compare samples, nodule characteristics, and proportion of unnecessary invasive approaches between the two periods. The Mann-Whitney test was used to compare mean age, mean number of diagnostic tests and time to diagnosis between periods. Results are expressed as percentages, means, and standard deviations (SDs). We tested the relation between number of diagnostic examinations and other variables in each period by multiple regression analysis. We used logistic regression models to identify the determinants of (1) invasive approaches in both periods, and (2) determinant factors for PET use for the after-PET period. A P < 0.05 was considered statistically significant for all analyses. The combination of SPN diagnostic tests in both periods was identified by use of SLPMiner, a data-mining algorithm for finding frequent sequential patterns [25,26] (i.e., time-ordered sequences of diagnostic tests performed for patients). The most frequent sequential patterns are identified as strategies and are presented as percentages. We described diagnostic test combinations that took place in 5% or more of cases in both periods.

The data were recorded in a Microsoft ACCESS database, and statistical tests involved use of SAS V 9.1 (SAS Inst., Cary, NC).

## Results

### Patient and nodule characteristics

**Figure 1:**
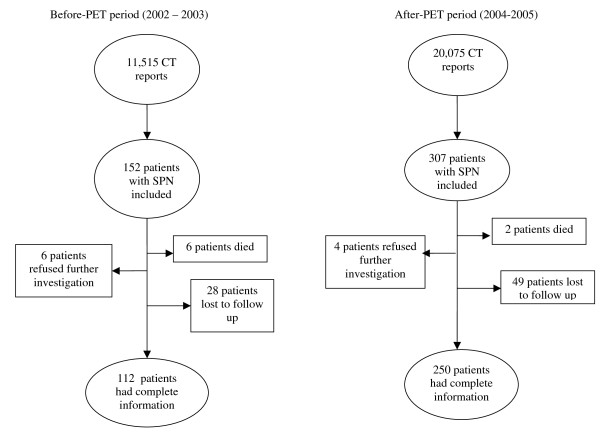
**The number of patients in both periods**.

A total of 459 patients with new cases of SPN (152 before-PET and 307 after-PET) were included. Of all patients included in both periods, 362 patients completed the study: 112 before-PET patients (73.7%), and 250 after-PET patients (81.4%). Eight patients died before a presumptive diagnosis, 10 patients refused further examinations, and 77 were lost to follow-up (Figure 1).

A greater proportion of patients with smoking history completed the study as compared with those who were lost to follow-up for both the before-PET (p = 0.002) and after-PET periods (p < 0.001). Patients whose diagnostic management was handled by specialists showed a more frequent tendency to have complete information for the study than those who were lost to follow-up in the after-PET period (p = 0.02). No other differences were observed in patient or nodule characteristics.

Patients in the before-PET and after-PET periods did not differ in age (mean 64.9 years [SE 14.1] *vs*. 64.8 years [SE 14.3]). Smoking habit differed between the two periods (58.4% for after-PET *vs*. 70.5% for before-PET; p = 0.03). Physicians tended to identify more calcified nodules and less spiculated nodules on CT results in the after-PET than before-PET period; the difference was not statistically significant (Table 1).

**Table 1:** Patient and nodule characteristics in the periods Before (2002–2003) and After (2004–2005) the availability of Positron Emission Tomography (PET)

Variable	Before-PET period		After-PET period		p
	
	Mean [SD]	n (%)	Mean [SD]	n (%)	
**Patient characteristics:**					
**Age**	64.9 [14.1]	112	64.8 [14.3]	250	0.9
					
**Age (in classes)**					
< 65 years		52 (46.4)		124 (49.6)	0.6
≥ 65 years		60 (53.6)		126 (50.4)	
					
**Sex**					
Male		82 (73.2)		158 (63.2)	0.06
Female		30 (26.8)		92 (36.8)	
					
**Referal**					
General practitioner		18 (16.1)		28 (11.2)	0.2
Specialist		94 (83.9)		222 (88.8)	
					
**Smoking history**					
Nonsmoker		33 (29.5)		104 (41.6)	0.03
Smokers or ex-smokers		79 (70.5)		146 (58.4)	
					
**Nodule characteristics:**					
**Calcified Nodules**					
Yes		12 (10.7)		45 (18.0)	0.08
No		100 (89.3)		205 (82.0)	
					
**Spiculated Nodules**					
Yes		32 (28.6)		49 (19.6)	0.06
No		80 (71.4)		201 (80.4)	
					
**Mediastinal node**					
Yes		31 (27.7)		75 (30.0)	0.7
No		81 (72.3)		175 (70.0)	

SD = standard deviation

### Management of SPN diagnosis

Patients in the before-PET period underwent a mean number of 4 tests before the presumptive definitive diagnosis of the nodule as compared to a mean of 3 tests in the after-PET period, but the difference was not statistically significant (p = 0.08). Table 2 describes mean number of tests according to patients and nodule characteristics in both periods. In the after-PET period, physicians prescribed a mean number of 5 tests for patients who underwent PET as compared to a mean of 3 tests for those who did not undergo PET (p < 0.001). Multivariate analysis showed that for both periods, patients who were referred by specialists and had spiculated nodules underwent more tests than patients who were referred by general practitioners and had nonspiculated nodule features (Table 3).

**Table 2:** Mean number of diagnostic tests prescribed in the periods Before and After the availability of Positron Emission Tomography (PET)

Variables	Before-PET period (n = 112)		After-PET period (n = 250)	
	
	Mean (SD)	p	Mean (SD)	p
**Patients**				
**Age (in classes)**				
< 65 years	3.8 (3.2)	0.6	3.5 (2.5)	0.04
≥ 65 years	4.7 (5.2)		2.9 (2.2)	
				
**Sex**				
Male	4.6 (4.8)	0.3	3.5 (2.5)	< 0.01
Female	3.5 (2.8)		2.5 (1.9)	
				
**Referal**				
General practitioner	2.2 (1.9)	< 0.01	1.7 (1.3)	< 0.01
Specialist	4.7 (4.6)		3.3 (2.3)	
				
**Smoking history**				
Nonsmoker	3.2 (4.2)	< 0.01	2.4 (1.9)	< 0.01
Smokers or ex-smokers	4.8 (4.4)		3.6 (2.5)	
				
**Nodules**				
**Calcified Nodules**				
Yes	3.1 (4.5)	0.049	2.2 (1.6)	< 0.01
No	4.5 (4.4)		3.4 (2.4)	
				
**Spiculated Nodules**				
Yes	5.3 (4.3)	0.048	4.9 (2.7)	< 0.01
No	3.9 (4.4)		2.7 (2.0)	
				
**Mediastinal node**				
Yes	4.5 (4.9)	0.9	3.7 (2.5)	< 0.01
No	4.3 (4.2)		2.9 (2.2)	

SD = standard deviation

p = Wilcoxon (Mann-Whitney) test

**Table 3:** Factors Iinfluencing the number of diagnostic tests patients with a Solitary Pulmonary Nodule underwent Before and After the availability of Positron Emission Tomography (PET) (n = 362)

Variables	Bivariate analysis		Multivariate analysis	
	
	β (SE)	p	β (SE)	p
**Period**				
Before-PET	-		-	
After-PET	-1.2 (0.4)	< 0.01	-0.96 (0.3)	< 0.01
				
**Age (in classes)**				
< 65 years	-		-	
≥ 65 years	-0.1 (0.3)	0.8	0.2 (0.3)	0.6
				
**Sex**				
Female	-		-	
Male	1.1 (0.3)	< 0.01	0.6 (0.4)	0.1
				
**Referal**				
General practitioner	-		-	
Specialist	1.8 (0.5)	< 0.01	1.8 (0.5)	< 0.01
				
**Smoking history**				
Nonsmoker	-		-	
Smokers or ex-smokers	1.4 (0.3)	< 0.01	0.5 (0.4)	0.2
				
**Calcified Nodules**				
Yes	-		-	
No	1.4 (0.5)	< 0.01	0.6 (0.4)	0.1
				
**Spiculated Nodules**				
Yes	-		-	
No	-1.9 (0.3)	< 0.01	-1.5 (0.4)	< 0.01
				
**Mediastinal node**				
Yes	-		-	
No	-0.6 (0.4)	0.09	-0.3 (0.4)	0.5

SE = standard error

Patients in the before-PET period had to wait 41.4 days, on average, before receiving a presumptive diagnosis as compared with 24.0 days, on average, for patients in the after-PET period who did not undergo PET (p < 0.001); those who underwent PET had to wait 56.7 days, on average, for a diagnosis. Bivariate analysis showed that patients who were male, were smokers, and had calcified and spiculated nodules were more likely to undergo PET. Multivariate analysis revealed that only spiculated nodules played a significant role in determining whether patients underwent PET (p < 0.001) (Table 4). The mean number of diagnostic tests in patients with spiculated nodules did not differ between the periods (5.3 for before-PET vs. 5.1 for after-PET; p = 0.4).

**Table 4:** Factors influencing the performance of Positron Emission Tomography (PET) in the period After PET was available (2004–2005) (n = 250)

Variable	Bivariate			Multivariate		
	**Odds Ratio**	**CI**	**p**	**Odds Ratio**	**CI**	**p**

**Age (in classes)**						
< 65 years	1		0.06	1		0.5
≥ 65 years	0.4	0.2 – 1.0		0.5	0.5 – 4.2	
						
**Sex**						
Female	1		0.04	1		0.1
Male	2.5	1.0 – 6		1.5	0.2 – 1.1	
						
**Referal**						
General practitioner	1		0.1	1		0.2
Specialist	4.7	0.6 – 35		3.7	0.5 – 29.7	
						
**Smoking history**						
Nonsmoker	1		< 0.01	1		0.4
Smokers or ex-smokers	3.9	1.5 – 9.7		1.6	0.5 – 4.6	
						
**Calcified Nodules**						
Yes	1		0.04	1		0.1
No	8.4	1.1–63		5.2	0.7 – 41.1	
						
**Spiculated Nodules**						
Yes	1		< 0.01	1		< 0.01
No	0.2	0.1 – 0.3		0.2	0.1 – 0.5	
						
**Mediastinal node**						
Yes	1		0.1	1		0.5
No	0.6	0.3 – 1.2		0.8	0.3 – 1.7	

CI = Confidence Interval

Logistic regression analysis of the probability of patients undergoing PET as one of diagnostic tests

Invasive approaches were more likely to be prescribed by specialists (p = 0.002) for smokers or ex-smokers (p < 0.001) with spiculated (p < 0.001) and noncalcified nodules (p < 0.001), but the proportion of patients undergoing at least one invasive test in the two periods did not differ (47% for before-PET vs. 49% for after-PET). In the after-PET period, of 185 patients with benign nodules, 54 (29.2%) underwent at least one invasive test, most frequently associated with PET (p < 0.001).

The proportion of patients undergoing CT of the thorax, bronchial fibroscopy, abdominal CT, and bone scintigraphy was almost similar in both periods. Less than 5% of patients in the after-PET period underwent lung scintigraphy, thoracotomy, and gastroscopy (Table 5). A total of 11% of patients underwent CXR as one of the diagnostic tests in the after-PET period as compared with 30% in the before-PET period (p < 0.001). A combination of CT of the thorax followed by bronchial fibroscopy was performed for almost the same proportion of patients in both periods (35.8% for before-PET vs. 33.7% for after-PET). However, a combination of abdominal echography and CT of the thorax was performed for less than 5% of patients in the after-PET period as compared with 16.7% in the before-PET period. In the after-PET period, physicians prescribed PET for 11% of patients, and 8% of them also underwent bronchial fibroscopy.

**Table 5:** Diagnostic tests performed in the periods Before and After the availability of Positron Emission Tomography (PET)

Type of diagnostic tests	Before-PET (112)		After-PET (250)	
	n	(%)	n	(%)
CT of the thorax	108	(96.7)	248	(99.2)
Bronchial fibroscopy	48	(42.5)	94	(37.5)
CT of the abdomine	37	(33.3)	79	(31.5)
CXR	34	(30.0)	27	(10.9)
CT of the skull	25	(22.5)	42	(16.9)
Abdominal echography	24	(21.7)	21	(8.5)
Bone scintigraphy	14	(12.5)	22	(8.9)
Transparietal ponction	12	(10.0)	15	(6.0)
Pulmonary scintigraphy	6	(5.0)	9	(3.6)
Thoracotomy	6	(5.0)	9	(3.6)
Gastroscopy	6	(5.0)	4	(1.6)
PET	0	-	27	(10.9)

Identified by use of SLPMiner. CT = computed tomography; CXR = chest x-ray

Of all patients included in the after-PET period, 62 (25.2%) had a diagnosis of cancer as compared with 30 (26.8%) in the before-PET period. Of 35 patients who underwent PET as one of their diagnostic tests, for 19 (54.3%), malignant nodules were revealed and for 11 (31.4%) no such diagnosis was made. Physicians prescribed PET to search for metastases in 4 patients (11.4%), and for 1 patient, PET was prescribed after the nodule was identified to be noncancerous.

Eighteen patients (29%) with a diagnosis of cancer underwent further lobectomy, bilobectomy or pneumonectomy for treatment, and these surgeries also served as diagnostic tests for 3 of the patients. Four patients underwent chemotherapy or radiotherapy in addition to surgery. Of all patients who received surgical treatments, one patient died about 1 year later due to brain, liver and lung metastases.

## Discussion

We found that the mean number of diagnostic tests performed for the management of SPN diagnosis declined after PET was introduced into the northeastern regions of France, but the reduction was not statistically significant. The mean number of diagnostic tests was fewer if prescribed by general practitioners and if the nodule was calcified or not spiculated. The reduction in mean number of diagnostic tests and time to presumptive diagnosis seemed to relate to study period rather than to use of PET. Physicians in the after-PET period seemed to prescribe less CXR and abdominal echography than in the before-PET period. This change was likely due to a generalisation and improvement of technology in that CT is considered more sensitive and specific than CXR in differentiating nodule status [1,9,27].

Even if the proportion of invasive tests performed did not differ between the two periods, unnecessary invasive approaches (i.e., invasive examination for patients with benign nodules) were less likely to be prescribed for patients who had undergone PET in the after-PET period. PET seemed to be prescribed when physicians considered a higher probability of malignancy of the nodule (spiculated) or to identify distal metastases. This finding confirmed the use of PET to differentiate malignant from benign nodules.

Both periods exhibited similar proportions of combinations of tests used. The management of SPN diagnosis for patients who underwent PET as one of their diagnostic tests in routine practice tended to confirm experimental conditions at the individual level. We found that 1 year after its availability, PET was not the immediate choice in daily practice, and therefore the frequency of PET prescription was quite low. Physicians tended to prescribe other techniques, even if invasive, that could be accessed immediately before prescribing PET, which was available only in the largest hospital in the region.

One study showed that PET as a noninvasive test was as efficacious as transthoracic aspiration needle biopsy in identifying malignant lung lesions and entailed no risk of complications [16]. Results of a retrospective Swiss study suggested that a combination of flexible bronchoscopy and PET had a useful role in the diagnosis of noncalcified chest lesions less than 3 cm seen on radiography and that bronchoscopy should be a first step because it allowed for a tissue diagnosis, was safe, and was more readily available [15]. Other studies of decision analysis models showed that CT-PET was the most cost-effective strategy in the management of SPN diagnosis [21,28]. In our study, 1 year after PET was made available, 11% of patients underwent this technology. One of the limitations of this study is that the onset of the after-PET observation period was too early and therefore the time interval for study was too short. Perhaps PET was not readily accessible for some patients because of the limited number of machines or the diffusion of information of the utilisation of PET was not fast enough to change physicians' habits in management of SPN diagnosis. However, like the Swiss study findings, in our study, PET was always performed at least after CT or after CT and bronchoscopy.

Results of many studies of PET performance in the diagnosis of SPN showed its superiority as compared with CXR or CT in differentiating malignant from benign nodules. Since randomizing patients to PET or non-PET groups is unethical, we decided to use a before-and-after design to evaluate any modification that might occur in the management of SPN diagnosis within participating regions. We considered a design that could also reduce a possible centre effect bias by incorporating all imaging centres in this large area.

We followed patients for 6 months after the presumptive diagnosis of their nodule considering that should the nodule's volume double in that period, the probability of its malignancy was high. Benign nodules are well known to have a longer doubling time than malignant nodules [12]. Some studies showed that for most malignant nodules, the volume doubling time is less than 400 days, with an average of 100 days in most cases [29-31]. Some studies suggested that the probability of nodule malignancy could be predicted from information on patient and nodule characteristics [17,32]. In daily practice, information on nodule size and its localisation, which could help physicians in the management of SPN diagnosis, was not always available on CT reports. Therefore, standardizing CT reports to include nodule characteristics (size, localisation, spiculation, calcification, and mediastinal node) would be useful.

Our study contained some limitations, such as a risk of measurement bias should any of the CT centres change instruments (CT camera) used to identify the SPN. To anticipate this potential bias, we asked each region to report any modification that might take place within or between the study periods. Some regions reported that new CT centres were opened between periods, and we included those centres as starting points to identify patients in the second period, but no major instrument changes were reported. Another limitation is that the after-PET period was probably still an implementation period in which prescription behaviours were not yet consolidated. Further observation after 3 years or more might allow for observing more use of PET.

Research studies showed that PET was more sensitive and more specific than other imaging techniques and more cost-effective than other strategies in diagnosing SPN [21,33,34]. However, in daily practice, PET did not directly contribute to the reduction in mean number of diagnostic tests or time to diagnosis. Its use did not reduce the frequency of unnecessary invasive examinations performed. In other words, use of PET brings clinical benefits to individual patients because of its performance, but at a population level, its benefits were not immediately observed and were of limited magnitude.

## Conclusion

This is the first study to evaluate the management of SPN diagnosis before and after the introduction of PET in a community. Despite the benefits that individual patients may experience, 1 year after the introduction of PET in oncology, clinicians do not yet allow a significant place for PET in the daily management of SPN diagnosis. Further study could be useful to evaluate the cost savings of actual modified practices and benefit for the health care system.

## Abbreviations

**PET: **Positron Emision Tomography; **SPN: **Solitary Pulmonary Nodule; **CT: **Computer Tomography; **CXR: **Chest X Ray; **CNIL: **Commission National d'Informatique et Liberté; **SD: **Standard Deviation; **SE: **Standard Error; **CI: **Confidence Interval.

## Competing interests

The authors declare that they have no competing interests.

## Authors' contributions

IL carried out the data analysis and interpretation, wrote and revised the manuscript; CB participated in data analysis and interpretation and in revising the manuscript; NJ participated in the data analysis, in particular using SLP Miner; KA participated in the study design, data analysis and interpretation; PA, DJ, MV, FV, and MC-WL participated in the study conception and design, and in revising the manuscript; CL participated in the study design and data interpretation; FG is the principal investigator of the study and participated in the study conception and design, data analysis and interpretation, and revision of the manuscript.

## Pre-publication history

The pre-publication history for this paper can be accessed here:

http://www.biomedcentral.com/1471-2407/9/139/prepub
